# Prospective study of urinary tract infection surveillance after kidney transplantation

**DOI:** 10.1186/1471-2334-10-245

**Published:** 2010-08-19

**Authors:** Roberto Rivera-Sanchez, Dolores Delgado-Ochoa, Rocio R Flores-Paz, Elvia E García-Jiménez, Ramon Espinosa-Hernández, Andres A Bazan-Borges, Myriam Arriaga-Alba

**Affiliations:** 1 Microbiology Research Laboratory, Hospital Juárez de México, Avenida Instituto Politécnico Nacional, México, D.F. 07760, México; 2 Histocompatibility Research Laboratory, Hospital Juárez de México, Avenida Instituto Politécnico Nacional, México, D.F. 07760, México; 3 Renal Transplant Surgery Division. Hospital Juárez de México, Avenida Instituto Politécnico Nacional, México, D.F. 07760, México; 4 Hospital Juarez De Mexico. Avenida Instituto Politécnico Nacional No. 5160, DF., 07760, México

## Abstract

**Background:**

Urinary tract infection (UTI) remains one of the main complications after kidney transplantation and it has serious consequences.

**Methods:**

Fifty-two patients with kidney transplantation were evaluated for UTI at 3-145 days (mean 40.0 days) after surgery.. Forty-two received a graft from a live donor and 10 from a deceased donor. There were 22 female and 30 male patients, aged 11-47 years. Microscopic examinations, leukocyte esterase stick, and urinary culture were performed every third day and weekly after hospitalization. A positive culture was consider when patients presented bacterial counts up to 10^5^ counts.

**Results:**

UTI developed in 19/52 (37%) patients at 3-75 days (mean 19.5 days after transplantation. Recurrent infection was observed in 7/52 (13.4%) patients at days 17-65. UTI was more frequent in patients who received deceased grafts compared with live grafts (7/10, 70% *vs*. 12/42, 28%; p < 0.007). Female patients were more susceptible than male (11/22, 50% *vs*. 8/22, 36.35%; p < 0.042). Five-year survival rate was 94.5% (49/52 patients). Kidney Graft exit update is 47/52 (90.2%), and there were no significant differences between graft rejection and UTI (p = 0.2518). Isolated bacteria were *Escherichia coli *(31.5%), *Candida albicans *(21.0%) and *Enterococcus *spp. (10.5%), followed by *Pseudomonas aeruginosa, Klebsiella pneumoniae, Morganella morganii, Enterobacter cloacae *and *Micrococcus *spp. Secondary infections were produced by (7/19, 36.8%). *Enterococcus *spp. (57%), *E. coli *(28%) and *Micrococcus *spp. (14.2%). Antibiotic resistance was 22% for ciprofloxacin and 33% for ampicillin. Therapeutic alternatives were aztreonam, trimethoprim-sulfamethoxazole, netilmicin and fosfomycin.

**Conclusions:**

Surveillance of UTI for the first 3 months is a good option for improving quality of life of kidney transplantation patients and the exit of graft function especially for female patients and those receiving deceased grafts. Antibiograms provided a good therapeutic alternative to patients who presented with UTIs after receiving a kidney allograft.

## Background

Treatment for patients with severe renal failure has been improved, especially with better surgical procedures and pharmaceutical management. Nevertheless, since these patients require immunosuppressive therapy, they are vulnerable to developing postoperative infections. Among these, urinary tract infections (UTI) are the most frequently observed and have high morbidity [1-3,5,6]. UTI was observed in 54.2% of 149 patients in Iran [7]. The frequency of UTI has varied between studies. A study in Tunisia has reported a UTI incidence of 43% within the first month and 33% for up to 6 months [8].**[ **In France, Pellé et al [9] have reported UTI in 77% of 177 patients who had undergone kidney transplantation. UTI after kidney transplantation has been associated with patient mortality and graft failure [10]. Nampoory et al [11] have recommended surveillance for UTI for a period of 6 months, to diminish the risk of renal failure. Charfeddine et al. [8] have reported that UTI was observed in all patients with graft rejection and 58% of patients without rejection.. Akinalow et al [12] have described UTI as an important risk factor for mortality in kidney transplantation patients.

Cytomegalovirus infections have been well documented as an important cause of graft rejection [7], therefore, recipients always receive antiviral therapy, before and after surgery for prophylaxis. In addition, bacterial infections have high morbidity and mortality and should not be discounted [7]. Several species of bacteria that cause UTI in kidney transplant patients have been isolated. *Escherichia coli *has been reported as the main uropathogen isolated in UTI among transplant patients in studies in Spain, India and Kuwait [11,13-15]. *Klebsiella *spp., *Pseudomonas *spp., including *P. aeruginosa*, and multiresistant isolates *of E. coli*, may by important etiological agents of these infections Gram-positive bacteria may also be important etiological agents of UTI, and the most frequently isolated have been *Enterocooccus *spp. and *Staphylococcus aureus *[4,11,14-17]. Less frequently, *Corynebacterium urealyticum *is considered as an etiological agent.

Infections caused by *Candida *spp. may be a serious problem in transplant recipients. They might cause infections of the bloodstream that can lead to sepsis. Such infection is caused frequently by catheter colonization and is improved with catheter removal. *Candida albicans *must be diagnosed rapidly because it may have fatal consequences [18,19].

Urinary culture, with microscopic examination and leukocyte esterase stick, has been recommended as a good predictor of symptomatic UTI [20]. Urinary culture in kidney transplant patients has been questionable, as a result of its cost, and it may be replaced with microscopic examination and the leukocyte esterase stick. Nevertheless, it is advisable to carry out urinary culture on kidney transplant patients within the first few months, because of their extreme vulnerability to UTI. Furthermore, urinary culture gives the opportunity of performing an antibiogram, which can lead to appropriate medical treatment. In fact, appropriate antibiotic therapy might give the patients a greater probability of preserving graft function [21,22].

This study aimed to establish the frequency of UTI in kidney transplant patients at Hospital Juárez de México, evaluate the greatest risk factors for developing UTI, and offer the physician and patient a good diagnosis and appropriate antibiotic therapy.

## Methods

### Study design

This was a prospective, clinical, noninvasive study. Fifty-two patients who underwent kidney transplantation at Hospital Juárez of México between November 1999 and October 2001 were included. Forty-two patients (22 female, 10 male, aged 11-47 years) received a graft from a living related donor and 10 from a deceased donor. A Foley catheter was installed before surgery, and it remains from 10 to 14 days afterwards. After surgery patients received an immunosuppressive treatment with prednisone, cyclosporine and azatioprine. All patients take as prophylactic treatment cefalosporine 2 generation 1.5 g/day for 10 days, aciclovir 400 mg and nitastatine 100 000 u each six hours for three months. Urinary samples were recovered directly from the catheter at 24-48 hours after surgery, and then every third day throughout the hospitalization period. Afterwards, patients were given sterile material and invited to take first morning sterile urine sample to the microbiology research laboratory each then days, when attending the Hospital for its usual medical control or cyclosporine detection levels at the HLA research laboratory.

### Ethical considerations

This study commenced following approval of the Research and Ethical Committees of the Hospital Juárez of México. Consent letters were not necessary because this study did not involve any invasive procedures for the patients. Patients were informed orally about the study and voluntary participation was offered after treatment of UTIs. The required sterile specimens were provided by the microbiology research laboratory and the cultures did not incur any financial costs for the patients.

### Urinary studies

Qualitative urinary cultures were done on blood agar, MacConkey agar and Biggy agar. as described previously [20] Cultures were incubated at 37°C for 24 hours. BiGGY plates were incubated for 72 hours. Urinary samples were evaluated with the leukocyte esterase stick, using Multistix 10 SG reagent strips (Bayer Diagnostics, S.A. de C.V., México, D.F.). Microscopic urinary sediment examinations were done after centrifugation of the sample at 1000 g for 15 minutes on a clinical centrifuge (Clay Adams Dynac, Beckton Dickinson Co.).[Parsippant N.J. 07054] Epithelial cells, urinary crystals and the number of leukocytes per microscopic field were recorded [20]. Urinary studies were performed every third day and weekly after hospital discharge. It was considered a positive result for Urinary tract infection [UTI], when bacterial counts were recorded up to 10^-5 ^counts, and leukocytes were up to 10 per microscopic field. Positive nitrates value was recorded for Gram negative bacteria. Lower bacterial counts were considered as bacteriuria, and they were not considered for the purpose of this study

### Susceptibility studies

Positive urinary cultures were processed for antimicrobial susceptibility testing on Mueller-Hinton agar plates, using the Kirby-Bauer disk diffusion method, according to the NCCLS specifications. Gram-negative bacteria were evaluated against ciprofloxacin, aztreonam, ampicillin, gentamicin, cefoperazone, arbenicillin, kanamycin, tetracycline, norfloxacin, aztreonam, trimethoprim-sulfamethoxazole, netilmicin and fosfomicin. *Enterococcus *spp. were evaluated against ampicillin, cefotaxime, penicillin, erythromycin, ceftriaxone, trimethoprim-sulfamethoxazole, and vancomycin. Susceptibility tests were performed employing commercial Sensi-Discs from BD-BBL, Becton Dickson Company Sparks, MD 21152, USA.

### Statistical analysis

Studies were evaluated with the *Χ*^2 ^test. Statistical analysis was performed with Graph-Pad version 2.01 software.

## Results

### Patient surveillance

Patients were evaluated for UTI from 3 to 150 days (mean 40.75 ± 40.0, arithmetic mode 40 days) after surgery. Four patients could not continue their evaluation beyond the first period as they lived outside Mexico City Other patients, continued voluntarily with microbiological evaluation for up to 3 months.

### UTI

**Figure 1:**
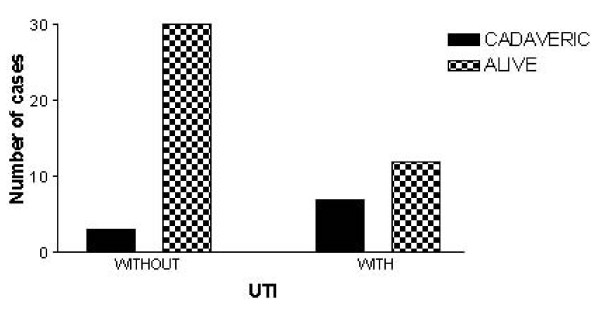
**Urinary tract infections with cadaveric or living donors**. Frequency of UTI among patients who received a deceased or live donor grafts. p = 0.007 (*Χ*^2 ^test)

UTIs were recorded in 19/52 (37%) patients. UTI was present as early as day 3 and as late as day 75, with a mean of 19.5 days. Reinfection was observed in 7/19 (36.8%) patients with previous UTI, from day 17 to 65. These infections were more frequent among patients who received a deceased graft compared with a graft from a live donor, as it is shown on Figure 1. Female patients were more susceptible to UTI than male patients (11/22, 50% *vs*. 8/30, 22%; p = 0.045). Forty-nine of 52 patients (94.5%) survived for up to 5 years. The graft exit monitoring up to 7 is descrived on (Table 1). Rejetion within UTI or not UTI, was higher, but these results were not statistically significant (p = 0.2518) (Table 2).

**Table 1:** Characteristics of patients with renal allograft.

*Characteristics of the evaluated group*	*Number of cases*	*Percentage*	***Significant Χ***^***2 ***^***test***
Women with UTI	11 [22]	50%	
Men with UTI	8 [30]	22%	p = 0.042
Deceased donor with UTI	7 [10]	70%	
Alive donor with UTI	12 [42]	28%	p = 0.007
Rejection with UTI	3 [33]	9%	
Rejection without UTI	2 [19]	10.5%	N.S. p = 0.46
Patient survival after 5 years	49 [52]	94.23%	
Graft function survival to present day	47 [52]	90.38%	
Death and UTI	0 [5]	0%	

**Table 2:** Patients with UTI and kidney allograft loss.

	*Allograft survival to present*	*Allograft rejection.*	(% )
With UTI	14	3	21.0%

**Without UTI**	33	2	6.06%

*Χ*^2 ^test; N.S. p = 0.1709

### Microbiological results

The first infections developed were caused mainly by *E. coli, C. Albicans*, *Enterocoocus *spp.*, and Enterobacteriaceae *(Table 3). The secondary infections were caused mainly by *Enterococcus *spp. (3/7, 47%), *E. coli *(2/7, 28.5%) and *Micrococcus *spp. (1/7, 14.28%).

**Table 3:** Isolated microorganisms from UTI from kidney transplant patients.

*Isolated strains*	*No. of cases*	*Frequency (%)*
E. coli	6	31.579

Candida spp.	4	21.053

Enterococcus spp.	2	10.526

A. calcoaceticus var. Anithratus	1	5.263

E. cloacae	1	5.263

K. oxytoca.	1	5.263

K. pneumoniae	1	5.263

M. morganii	1	5.263

P. aeruginosa	1	5.263

Micrococcus spp.	1	5.263

Total	19	100.000

### Antimicrobial agents

The most recommended antibiotics for kidney transplant patients were ciprofloxacin and ampicillin. Ciprofloxacin resistance was observed among 22% of isolated strains, and ampicillin resistance among 33% of the isolated Gram-negative bacteria. Therapeutic alternatives for these cases were aztreonam, trimethoprim-sulfamethoxazole, netilmicin and fosfomycin. A multiresistant *Enterococcus *was recovered, which was sensitive only to vancomycin.

## Discussion

This work demonstrated that, despite prophylactic treatment of kidney transplant patients, UTI was a major postoperative complication. Nineteen of 52 patients (37%) developed at least one episode of UTI. These results are similar to those of Kanisauskaite et al [23], who reported UTI in 37% of 57 patients, and Memikoglu [4], who found UTI in 41% of 136 patients in Turkey. However, these numbers of patients are lower than those reported by Poumard et al [7], who found UTI in 54% of 179 patients in Iran. These works state up the need of including studies of infectious diseases besides the renal function tests after kidney transplantation.

In the present study, we found that patients who received a deceased graft were more susceptible to UTI, compared with a graft from a living related donor (70% *vs*. 28%), and may reflect an asymptomatic infection in the cadaver donor which developed in the immunosuppressed patient; in contrast, live donors are evaluated carefully before surgery. Similar results have been reported by Midtvedt et al [24], who reported that deceased kidney recipients had higher rates of infections. In the present study, female sex was found to be a risk factor for UTI, as reported previously [4].

It has been proposed that surveillance of UTI in kidney transplant patients should be performed over a long period. Kumar et al [21] have suggested evaluation over the first 100 days, and Dupont et al [25] have reported that late UTI may damage renal allografts at more than a year after surgery. We found UTI between 3 and 75 days after surgery, therefore, we confirm that surveillance should be carried out for 3 months after kidney transplantation.

Our patients have continued to the present day with evaluation of cyclosporine levels at the HLA research laboratory, as well as medical evaluation. The 5-year survival was 94.5% (49/52 patients), and 47 patients have survived to the present. The graft exit up to seven years was 47/52 [90.2%]. This result is higher than that reported by Kanisauskaite et al [23] in Lithuania, who had 85% of kidney survival exit.

In our patients, *E. coli *was the main agent that caused UTI after kidney transplantation,[table 3] and the infective agent in one of the three patients who suffered kidney allograft rejection, which is similar to several other studies from around the world [4,11,14,15,19]. The second major cause of infection in our patients was *C. albicans*, which was cultured from four patients, two of whom developed reinfection with *Enterococcus *spp. and *E. coli. C. albicans *infection in our patients was more common than that observed by Valera et al in Spain [15]. *Candida *infection can have serious consequences if it is not detected early. Catheter removal and amphotericin B is a good therapeutic option for these patients [18]. Unfortunately, one of the four patients infected with *Candida *spp. suffered graft rejection before day 10.

*S. aureus *is one of the most important microorganisms that causes UTI in transplant patients but it was not observed in the present study [26,27]. Among the Gram-positive bacteria, the main organism observed in our study was D *enterococci*, both in primary and secondary infections. One of the patients infected with this bacterium suffered allograft rejection before day 10. These results are in agreement with previously published studies. Alangaden [13] and Leigh et al. [26], have designated *Enterococcus *spp. as an emerging bacterium that causes symptomatic infections, especially in kidney transplant patients.

The cost-benefit of urinary cultures has been questioned, especially in asymptomatic UTI in kidney transplant patients [28]. It is thus advisable to conduct additional studies to evaluate the advantages of performing cultures in all post-transplant patients for UTI surveillance.

In contrast, it has been reported that multiresistant bacteria might be cultured from kidney transplant patients as a consequence of prophylactic therapy, therefore antibiograms of the infecting microorganisms have been suggested [1,3,16]. Antibiograms were performed for infective bacteria in our study, in order to offer more appropriate therapy to patients. Antibiograms in these patients are useful to reduce employment of antibiotic therapy on unnecessary cases and to improve antibiotic therapy on kidney transplanted patients. Twenty-two percent of our isolates were resistant to ciprofloxacin, which is used widely in kidney transplant patients, although this was lower than the 50% resistance reported by Senger et al. in 2007 [3]. In the present study, ampicillin resistance was observed in 33% of the Gram-negative strains. The main therapeutic options were aztreonam, trimethoprim-sulfamethoxazole, netilmicin and fosfomycin. Cephalosporin showed intermediate resistance in our study, whereas, Lazinzka et al [29] reported that 90% of Gram-negative strains isolated from kidney transplant patients in Poland were susceptible to ceftriaxone and ceftazidime. These result might explain the failure of the employed prophylaxis in patients developing UTI. In fact, it is known that antibiotic resistance is a frequent medical problem due to the injudicious use of these drugs; however an individual antibiogram, as obtained in this study, gave a good therapeutic alternative to patients who presented with UTI after receiving a kidney allograft.

Surveillance of UTI in the first 3 months after surgery, using urinary culture, leukocyte esterase stick and antibiograms, is a good way to reduce the risk of UTI in transplant patients and the exit of the graft function, especially those receiving deceased grafts or female patients. In the present study, 3/19 patients with UTI suffered kidney graft rejection, while only 2/33 patients without UTI had graft rejection. This difference was not statistically significant, which differs from previous studies that have stated that UTI might be a cause of graft rejection [30]. Nevertheless, it may be worthwhile to advise patients to continue with their clinical and bacteriological evaluation after surgery.

## Conclusions

Surveillance of UTIs for the first 3 months is a reasonable option for improving graft function free of kidney infections and assuring the quality of life for the kidney transplant population and the loss of graft function, especially for female patients and those receiving suspicious deceased grafts. Antibiograms give a good therapeutic alternative to patients who present with UTIs after receiving a kidney allograft.

## Competing interests

All of the authors confirm that do not have any competing interests or financial gain or commercial compromise based on the work in this investigation.

## Authors' contributions

RRS carried out the bacteriological studies and antibiograms; DDO performed cyclosporine surveillance and post-hospital interviews; RFP and EGJ performed the urinary cultures and microscopic examinations; ABB and REH carried out the kidney transplant surgery; REH has revised the manuscript. MAA designed the study, performed the statistical analysis and prepared the manuscript. All authors read and approved the final manuscript.

## Pre-publication history

The pre-publication history for this paper can be accessed here:

http://www.biomedcentral.com/1471-2334/10/245/prepub
